# Socioeconomic inequality in treatment of acute myeloid leukaemia (AML) in England, 2015–2022: A population‐based study

**DOI:** 10.1111/bjh.70695

**Published:** 2026-07-14

**Authors:** Koki Shimizu, Mari Kajiwara Saito, Camille Maringe, Yasushi Miyazaki, Bernard Rachet

**Affiliations:** ^1^ Inequalities in Cancer Outcomes Network, Department of Health Services Research and Policy, Faculty of Public Health and Policy London School of Hygiene and Tropical Medicine London UK; ^2^ School of Tropical Medicine and Global Health Nagasaki University Nagasaki Japan; ^3^ Department of Haematology Atomic Bomb Disease Institute, Nagasaki University Nagasaki Japan

**Keywords:** acute myeloid leukaemia, England, NHS Trust, socioeconomic inequalities

## Abstract

The socioeconomic inequality in acute myeloid leukaemia (AML) survival may be driven by differential access to intensive chemotherapy (ICT). We aimed to quantify socioeconomic inequality in the receipt of ICT among patients with AML in England and identify its key drivers. We evaluated 10 495 patients with de novo AML identified in the national cancer registry of England (2015–2022) along with their area‐deprivation status, as a measure of socioeconomic inequality, and treatment information based on linkage databases. We related prevalence of the ICT receipt to deprivation with multivariable‐adjusted generalised regression and mixed‐effects probit regression. Overall, 5190 patients (49%) received ICT. The patients in the most deprived area were 6.1% less likely to receive ICT than those in the least deprived area. The differences in the prevalence measures varied across the 125 administrative clusters of health service units. If all patients had access to ICT similar to the least deprived patients, 247 more patients would receive ICT. Of the deprivation effect, 94% was estimated to be attributable to the variability among administrative clusters. Despite universal healthcare coverage, socioeconomic inequality exists in AML treatment in England. The inequality may be driven by cluster‐level variations such as institutional culture and resource capacity.

## INTRODUCTION

The prognosis of acute myeloid leukaemia (AML) remains dismal for older patients. According to a regional registry in England, the 5‐year relative survival of AML is 16% in patients aged between 60 and 69 and 5% in patients aged between 70 and 79.^1^ AML is most common among older patients, yet their treatments are further complicated by comorbidities and unfavourable genetic mutations.^2^ A population‐based study from the Netherlands, which has universal healthcare coverage (UHC), reports a regional variability in application of intensive chemotherapy (ICT) for older patients, although it can potentially yield long‐term survival in fit and eligible older patients.^3^

The administration of AML treatment is not only determined by patients' age and comorbidities but also by their socioeconomic status. In the United States, which does not have UHC, socioeconomic factors such as household income, type of insurance and race/ethnicity were associated with AML treatment receipts and survival.^4^, ^5^ Socioeconomic inequalities in treatment also exist in countries with UHC. In a Danish population‐based study, older patients with low education received ICT less than those with high education.^6^ Consequently, the survival improved only among the high education group. Survival disparities based on socioeconomic status are similarly reported from Sweden, Germany, France and England, which all have UHC.^7^, ^8^, ^9^, ^10^

Previous literature suggests that socioeconomic inequalities in AML survival exist, yet few studies quantified inequalities in treatment and investigated the driving mechanism of such inequality in the context of UHC. These are crucial in informing the stakeholders, designing more equitable services and ultimately addressing the deprivation‐related survival gap.^10^ This study aimed to quantify socioeconomic inequality in ICT receipt among patients with AML in England and to identify drivers of this inequality.

## METHODS

### Study population

This was a nationwide, observational study using the National Cancer Registration Dataset (NCRD) of England. Patients were all entitled to free medical care under the National Health Service (NHS). NHS Hospitals were organised into operationally distinct legal entities known as trusts. An NHS trust consisted of one or a group of hospitals that served a geographically defined catchment area.^11^

We evaluated patients aged 25–80 years, newly diagnosed with de novo AML between 1 January 2015 and 31 December 2022, who were followed until death, or for a minimum of 1 year since diagnosis. We identified incident cases of AML in the NCRD using the International Classification Diseases for Oncology, 3rd Edition (ICD‐O‐3) morphology codes (Table S1).^12^ The NCRD is systematically collected across all health institutions funded by the NHS through the National Disease Registration Service (NDRS) and has been anonymised.^13^

We excluded patients with acute promyelocytic leukaemia because its treatment differs from other AMLs. We also excluded secondary AMLs (s‐AMLs): antecedent haematological disorder or if they had received chemotherapy or radiotherapy. We chose to exclude s‐AML because we could not obtain the information of the disease status of the previous malignancy and the status of organs after being exposed to cytotoxic treatments (e.g. anthracycline cardiotoxicity), which may affect treatment choice. We retrieved the conditions of s‐AML from the NCRD, the Hospital Episode Statistics (HES) and the Radiotherapy Dataset (RTDS) (Tables S2 and S3). The HES contained records of inpatient admissions and outpatient appointments at NHS hospitals with relevant diagnosis and procedure codes.^14^ The RTDS provided information on radiotherapy delivery at acute NHS hospitals.^15^

### Deprivation

We defined patients' socioeconomic status based on the Income Domain of the Index of Multiple Deprivation 2019, calculated by the Ministry of Housing, Communities and Local Government.^16^ It measured the proportion of the population receiving social security benefits related to low income in an area.^17^ A relative deprivation score was assigned to 32 844 small areas across England; on average, each area consisted of 1500 residents or 650 households. The level of deprivation was expressed as quintiles from Q1, least deprived, to Q5, most deprived, and was linked to patients in the NCRD based on their home address at the time of diagnosis.

### Outcome

**FIGURE 1:**
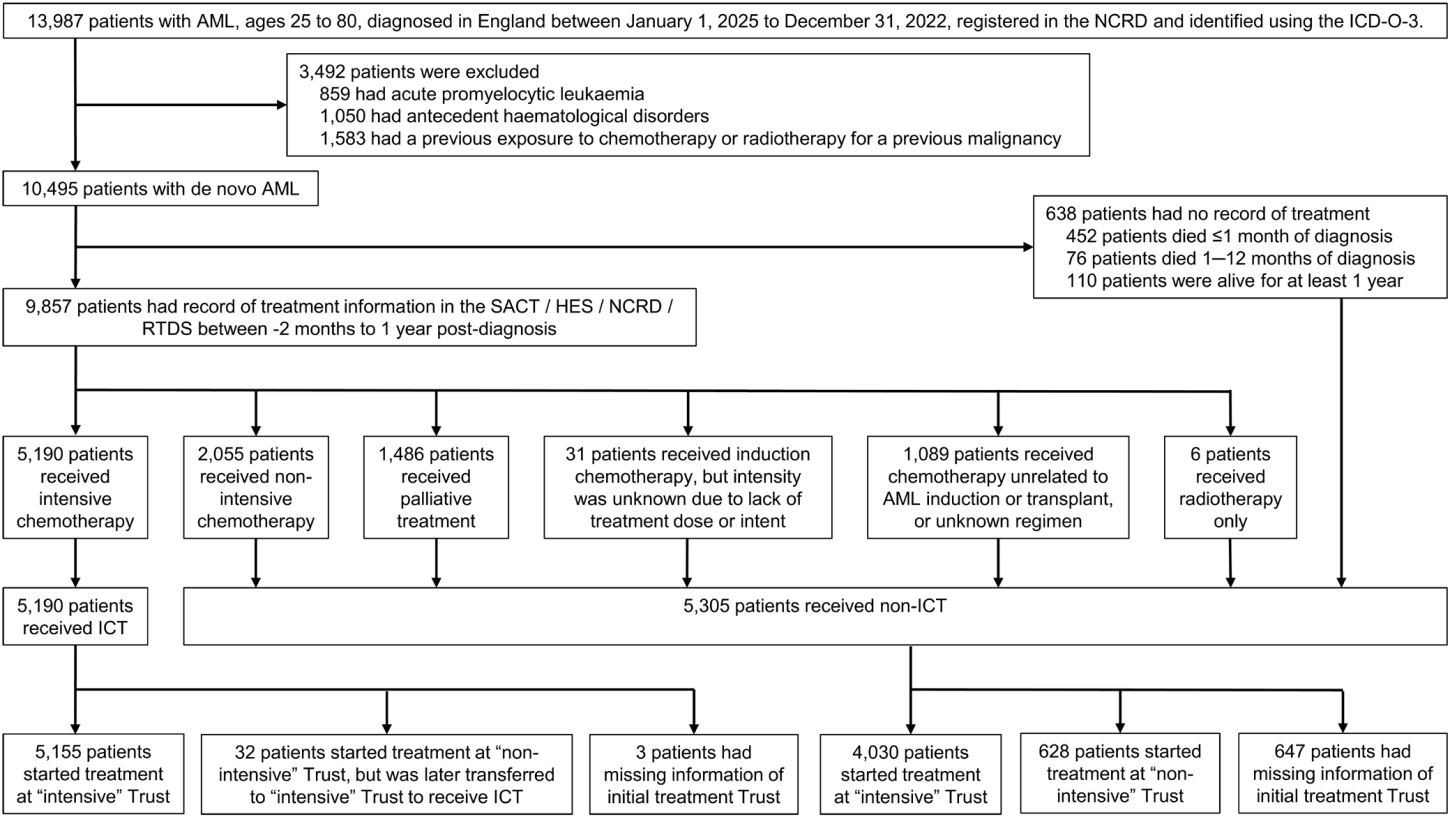
A flow chart of patients from identification to types of treatment. The flow chart describes the number of patients from identification to their types of treatment. AML, acute myeloid leukaemia; HES, Hospital Episode Statistics; ICT, intensive chemotherapy; NCRD, National Cancer Registration Dataset; RTDS, Radiotherapy Dataset; SACT, Systemic Anti‐Cancer Therapy.

The primary outcome was the receipt of ICT (Figure 1). We retrieved the treatment information of individual patient by linking the NCRD to other three national datasets, the Systemic Anti‐Cancer Therapy dataset (SACT), the HES and the RTDS, using unique identifiers. The SACT captured information on anti‐cancer therapies, including chemotherapy, administered in all NHS hospitals in England.^18^ We performed the linkage of datasets in a hierarchical order from the SACT, the HES and the RTDS (Appendix S1, Figure S1, Tables S4─S6). We searched for treatment information from the period between 2 months prior to AML diagnosis up to 1 year post diagnosis (Figure S2).

The lead author (K.S.) made a decision on the intensity of treatment, which was reviewed by two local haematologists. We defined patients with ICT as those who had a record of at least one cycle of intensive induction chemotherapies (e.g. daunorubicin and cytarabine) or allogeneic haematopoietic stem cell transplantation (HSCT) regimens during the treatment period. We defined other treatments (e.g. azacitidine), palliative treatments, chemotherapy unrelated to AML induction or HSCT, unknown regimen, radiotherapy or no record of treatment as non‐ICT. If patients started with a non‐ICT induction regimen, but later received HSCT, then they were classified as ICT.

We identified 125 NHS Trusts where patients initiated their treatments. We labelled trusts as ‘intensive’ if they had the capacity to provide ICT and ‘non‐intensive’ if they did not have the capacity to provide ICT.^19^

### Variables

Demographic variables included age at diagnosis and sex, derived from the NCRD. We defined comorbidity conditions as listed in the Charlson Comorbidity Index and searched for the corresponding ICD‐10 codes in the NCRD and the HES (Table S7).^20^

### Statistical analysis

We estimated the average, age‐specific and trust‐specific prevalence of ICT receipt across deprivation quintiles. For all our analyses, we considered patients' NHS Trust of initial treatment as a cluster.

To estimate the average and age‐specific prevalence of ICT receipt, we used a generalised estimating equation model with exchangeable correlation structure and robust variance estimator. We used a probit link function to estimate the effect of deprivation on ICT receipt. To estimate trust‐specific prevalence of ICT receipt, we used a mixed‐effects probit regression model.

In all our analyses, we adjusted for the confounding effect of age, sex and comorbidities. We treated age as a continuous variable and assumed a non‐linear association between age and ICT using flexible functional forms (restricted cubic splines with knots at age 60, 65 and 70). We tested for an interaction between age and deprivation because the association between deprivation and ICT receipt may vary by age. We treated comorbidities as a categorical variable (0, 1, 2 or more).

We investigated a presence of interaction between deprivation and calendar year periods (2015–2019 vs. 2020–2022) to account for the potential change in leukaemia services during the coronavirus disease‐19 (COVID‐19) pandemic. We also examined the presence of interaction between deprivation and urbanicity (≤30 min travel by road to a major town or city vs. >30 min).^21^

We tested the strength of association between deprivation and ICT receipt and the presence of interactions using a joint Wald *χ* ^2^ test.

All statistical analyses were performed using STATA, version 18.0 (StataCorp, College Station, TX, USA).

### Ethical approval

The data for this study were collected by the NDRS under the National Disease Registries Directions 2021 from the Secretary State for Health and Social Care, under section 254 of the Health and Social Care Act 2012.^22^ The use of data has been approved by NHS Health Research Authority London—Central Research Ethics Committee (REC reference: 21/LO/0552; IRAS project ID: 279592) and this study protocol by ethical committee of the London School of Hygiene and Tropical Medicine (reference: 31448).

## RESULTS

A total of 10 495 patients, aged between 25 and 80, were newly diagnosed with de novo AML between 2015 and 2022 in England (Figure 1). The median age at diagnosis was 67 years, and 58% were male (6079/10495) (Table 1).

**TABLE 1:** Baseline characteristics of patients with AML aged between 25 and 80, diagnosed in England between 2015 and 2022, stratified by deprivation (*N* = 10 495).

	Total	Q1 (least deprived)	Q2	Q3	Q4	Q5 (most deprived)
	*N* (%)	*N* (%)	*N* (%)	*N* (%)	*N* (%)	*N* (%)
Patients^a^	10 495	2174 (21)	2248 (21)	2228 (21)	2035 (19)	1810 (17)
Sex						
Male	6079 (58)	1269 (58)	1323 (59)	1296 (58)	1144 (56)	1047 (58)
Female	4416 (42)	905 (42)	925 (41)	932 (42)	891 (44)	763 (42)
Age (years)						
Median (IQR)	67 (56─74)	69 (59─75)	69 (57─75)	67 (57─74)	66 (55─73)	64 (52─73)
25─59	3209 (31)	574 (26)	590 (26)	652 (29)	684 (34)	709 (39)
60─69	2811 (27)	554 (25)	580 (26)	636 (29)	566 (28)	475 (26)
70─80	4475 (43)	1046 (48)	1078 (48)	940 (42)	785 (39)	626 (35)
Number of comorbidities						
0	7119 (68)	1549 (71)	1592 (71)	1482 (67)	1353 (66)	1143 (63)
1	2076 (20)	408 (19)	426 (19)	453 (20)	392 (19)	397 (22)
2 or more	1300 (12)	217 (10)	230 (10)	293 (13)	290 (14)	270 (15)
Receipt of intensive chemotherapy						
Yes	5190 (49)	1072 (49)	1103 (49)	1107 (50)	998 (49)	910 (50)
No	5305 (51)	1102 (51)	1145 (51)	1121 (50)	1037 (51)	900 (50)

Abbreviations: AML, acute myeloid leukaemia; IQR, interquartile range.

^a^ First row of the table is in row proportion, and the remaining of the table is in column proportion.

Treatment information was available for 94% of the cohort (9857/10 495) (Figure 1). Forty‐nine per cent of patients (5190/10 495) received ICT, with a clear decline by age at diagnosis, from 82% (2631/3209) of 25–59 years to 63% (1765/2811) of 60–69 years and 18% (794/4475) of 70–80 years.

### ICT prevalence and deprivation

After adjusting for age, sex and comorbidity, increased deprivation was associated with lower prevalence of ICT receipt (Table 2). On average, the most deprived patients (Q5) were 6.1% less likely to receive ICT than the least deprived patients (Q1) (difference in prevalence: −6.1%, 95% confidence interval [CI]: −8.8% to −3.4%). The COVID‐19 pandemic and urbanicity did not modify the association between deprivation and ICT receipt (p‐value for interaction: COVID‐19 = 0.80, urbanicity = 0.85). On the contrary, there was modest evidence that age modified the association (p‐value for interaction = 0.21).

**TABLE 2:** Difference in prevalence of ICT receipt among patients with AML, in ages between 25 and 80, diagnosed in England between 2015 and 2022 (*N* = 9845).^a^

	Crude difference in prevalence (%)	95% confidence interval (%)	*p*‐value^b^	Adjusted difference in prevalence (%)	95% confidence interval (%)	*p*‐value^b^
Q1 (least deprived)	Reference		0.70	Reference		<0.001
Q2	0.4	(−2.4, 3.1)		−0.4	(−2.7, 2.0)	
Q3	0.7	(−1.8, 3.3)		−2.6	(−4.6, −0.5)	
Q4	−0.3	(−3.4, 2.7)		−5.7	(−7.8, −3.6)	
Q5 (most deprived)	1.6	(−1.6, 4.8)		−6.1	(−8.8, −3.4)	

Abbreviations: AML, acute myeloid leukaemia; GEE, generalised estimating equation; ICT, intensive chemotherapy.

^a^ We analysed 9845 patients who had information of the initial treatment trust. The multivariable GEE model adjusted for age, sex and comorbidity and included an interaction between age and deprivation.

^b^ *p* values of joint Wald *χ* ^2^ test.

Adjusting for comorbidity did not alter the association between deprivation and ICT receipt. The difference in the prevalence of ICT receipt between Q1 and Q5 was large in ages between 60 and 70 (Figure S3).

### ICT, deprivation and trusts

Overall, 88% of patients initiated treatment at intensive trusts where ICT could be performed (9185/10495). The proportions of patients who initiated treatment at intensive trusts by deprivation levels were similar in ages between 25 and 69 (Table S8).

**FIGURE 2:**
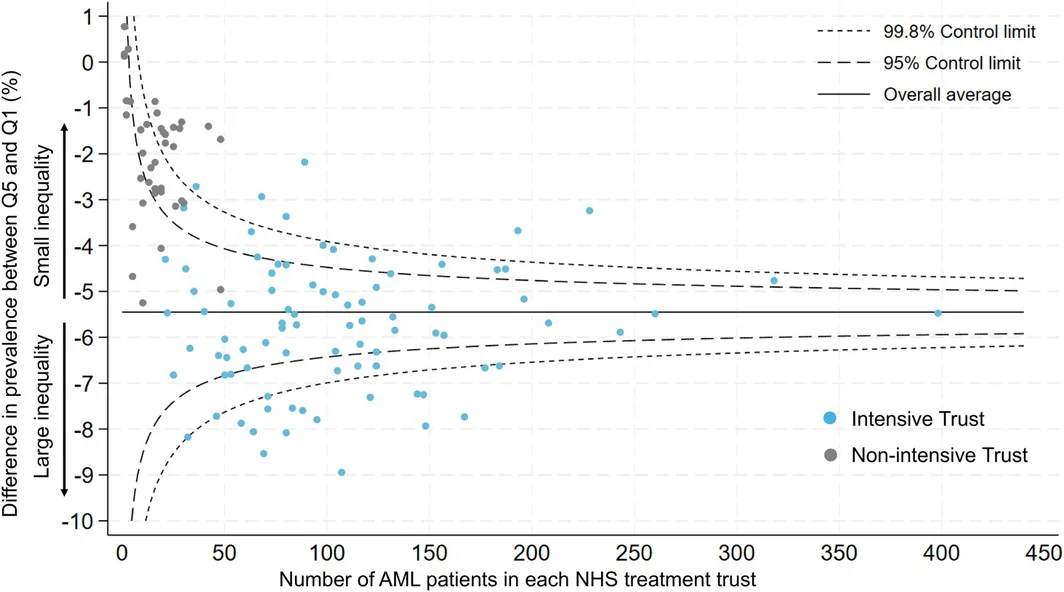
Difference in prevalence of ICT receipt between Q1 and Q5 at each NHS Trust^a^. The funnel plot described the difference in prevalence of ICT receipt between Q1 and Q5 at each NHS Trust where patients initiated treatment. We used mixed‐effects probit regression model to predict the adjusted prevalence, while treating NHS Trust as a cluster. The inequalities between Q1 and Q5 were smaller when patients initiated treatment at non‐intensive trusts than when they initiated treatment at intensive trusts. The large inequality was observed among intensive trusts of intermediate size (patient volume of 50–200). ^a^The analysis included 9845 patients who had information of the NHS Trust where they initiated treatment. ICT, intensive chemotherapy; NHS, National Health Service.

The difference in the prevalence of ICT receipt between Q1 and Q5 varied by trusts (Figure 2). The differences between Q1 and Q5 were small if patients started treatment at non‐intensive trusts where ICT could not be performed. The differences were large if they started treatment at intensive trusts of intermediate size (patient volume of 50–200 patients).

### Predicted number of patients with ICT

If we assumed that all patients had access to ICT similar to Q1, but remained in their original trust, the model predicted that 16 more patients (out of 3668 patients) would potentially receive ICT (Table 3). If we assumed that all patients had access to ICT similar to Q1 and were uniformly treated under standardised care across trusts, the model predicted that 247 more patients would potentially receive ICT.

**TABLE 3:** The predicted prevalence of patients with ICT based on the original distribution of deprivation and in a hypothetical model where deprivation is switched to Q1 (*N* = 9845)^a^.

Predicted prevalence of ICT (original deprivation)^b^	Predicted prevalence of ICT (deprivation switched to Q1, but patients remained in their original NHS Trust)^b^	Increase in prevalence	Predicted prevalence of ICT (deprivation switched to Q1, all patients treated in the NHS Trust in a uniform manner)^c^	Increase in prevalence	Proportion of deprivation effect explained by trust‐level variability
52.7% (*N* = 5184)	52.8% (*N* = 5200)	+0.1%	55.2% (*N* = 5431)	+2.5%	93.5%

Abbreviation: ICT, intensive chemotherapy.

^a^ The analysis included 9845 patients who had information of trusts where they initiated treatment.

^b^ Mixed‐effects probit regression was used to estimate the predicted numbers where NHS Trust was treated as a cluster.

^c^ Fixed‐effect probit regression was used to estimate the predicted numbers, accounting for case‐mix (age, sex, comorbidity).

## DISCUSSION

### Key findings

This population‐based study of England suggested that more deprived patients were less likely to receive ICT than less deprived patients, after adjusting for age, sex and comorbidities. This treatment inequality persisted during the pandemic. The treatment inequality between the most and the least deprived patients was large between ages of 60 and 70, regardless of sex and comorbidity status. It also varied by NHS Trusts; the large treatment inequality was observed among intermediate‐volume intensive trusts.

### Comparison with existing literature

Our findings were not consistent with a population‐based study in Denmark that reported no significant association between deprivation and receipt of ICT.^6^ Importantly, both England and Denmark had UHC; socioeconomic deprivation would not influence healthcare access as strongly as in private healthcare settings. The Danish study suggested that low education was associated with lower receipt of ICT among older patients. Similarly, our results indicated that the deprivation‐related treatment inequality was large in patients aged 60–70 years at diagnosis. This disparity may result from an institutional variation in the administration of ICT among older patients, where treatment decisions are complex.^3^ ICT can confer long‐term survival benefits in fit and eligible older patients, but the assessment of patient fitness and eligibility is multifactorial, considering both the biological and social conditions. The implementation of ICT will necessitate additional clinical support as well as proactive engagement by the patients and their families. These may not be readily available for those from deprived backgrounds. Additionally, treatment options for older adults diversified after the approval of venetoclax in 2022 by the National Institute for Health and Care Excellence. The clinical decision‐making became a more fine‐grained judgement between ICT; more effective non‐ICT such as a combination therapy of venetoclax and azacitidine; and conventional non‐ICT such as low‐dose cytarabine and azacitidine alone.

### Implications

The persistence of the socioeconomic inequality during the pandemic suggested that universal constraints of health services did not affect treatment inequality. Before the pandemic, the prevalence of ICT was 51% (3402/6679); after the pandemic, the prevalence of ICT dropped to 47% (1788/3816). The decline in the prevalence of ICT affected all deprivation quintiles; the more deprived patients remained less likely to receive ICT than the less deprived patients, even when NHS cancer services were highly restricted.^23^

A similar proportion of the most and the least deprived patients in ages between 25 and 69 initiated treatment at intensive NHS trusts. This suggests that the final treatment decisions for these groups were likely made by clinicians at intensive trusts. Therefore, the observed treatment inequality does not appear to be driven by lower referral rates or reduced access to intensive trusts among the more deprived patients. More detailed information on referral pathways including the sequence and timing of key events and patient status at each time point are needed to better understand the differential impact of pretreatment factors on the treatment decisions at intensive trusts.

The treatment inequality between the most and the least deprived patients was small in non‐intensive trusts. For inequality to occur among the non‐intensive Trusts, patients needed to be transferred to intensive trusts to receive ICT after initiating treatment at non‐intensive trusts. The data indicated that such transfers were not common. The patients in non‐intensive trusts were older and had more comorbidities than those in intensive trusts (Table S9); they may have been referred to non‐intensive trusts because they were deemed less eligible for ICT by the clinicians.

On the contrary, the large treatment inequality was observed among patients who initiated treatment at intensive trusts; specifically, the inequality was larger for intermediate‐volume intensive trusts than large‐volume intensive trusts. The intermediate‐volume trusts could perform ICT like large‐volume trusts, but may not have the resources and the capacity as large‐volume trusts (i.e. national leukaemia centres). The large‐volume trusts may provide services such as transportation between home and hospital that could potentially narrow the deprivation gap. The comparison between the intermediate‐ and the large‐volume intensive trusts may provide a clue to understand the root of deprivation‐related treatment inequalities.

The effect of deprivation on ICT receipt was attenuated after adjusting for between‐trust variability. This suggests that deprivation‐related inequality in treatment may not be driven by deprivation itself, but by variations between trusts in terms of AML services. Variations in clinical outcomes at the trust level are well documented, and they may be a reflection of differences in pretreatment assessment, clinical practice, toxicity monitoring and the availability of infrastructure.^24^ We may be able to understand the cause of such variation by investigating the factors that influence decision‐making within each trust, including institutional culture, workforce capacity and resource availability.

### Strengths and limitations

A major strength of this study is the use of the most recent available data from the NCRD—a high‐quality, population‐based dataset that covers as high as 99% of patients with cancer in England.^25^ The SACT dataset does not include chemotherapy administered in private hospitals; because AML treatment is rarely delivered in private settings (<1%), this has a small impact on our findings. Importantly, patients from socioeconomically deprived backgrounds are often underrepresented in clinical trials and published studies.^26^ This real‐world analysis, therefore, provides a more complete picture of treatment patterns across the socioeconomic groups in England. The 8‐year study period, which includes the recent period after the COVID‐19, further enhances the relevance of the findings to current NHS cancer service delivery.

This study has several limitations. First, we used an area‐level deprivation as a measure of patients' socioeconomic status. We recognise that both contextual and individual‐level deprivation may pose different obstacles in healthcare access and usage. We could only measure the effect of contextual deprivation.^27^ Second, the routinely collected datasets are subject to misclassification and missing data. For example, despite improvements from data linkage, some s‐AML cases may have been misclassified as de novo AML due to missing records of prior haematological diagnoses or cytotoxic exposure. Patients with s‐AML typically have higher comorbidity and poorer treatment response, and their inclusion may affect observed treatment patterns.^28^ However, s‐AML was more common among older adults, and patients in Q1 were older than Q5. Therefore, this misclassification would likely bias the results towards the null, underestimating the true inequality in ICT receipt. Third, patient‐level factors such as the genetic risk of AML may confound the association between deprivation and ICT receipt. The distribution of cytogenetic risks was reported to be similar across deprivation levels in a Danish population‐based study.^6^ Fourth, this study did not assess the effect of patients' fitness and late presentation on ICT receipts. A sensitivity analysis on 50% of the cohort with information of performance status, as a proxy for fitness, indicated that the effect of deprivation may be partially explained by difference in performance status (Table S10). However, cases with information of performance status may not represent the entire cohort. Performance status at the time of treatment initiation may not accurately reflect the baseline fitness of the patients. The study did not assess the role of late presentation because we did not account for the timing of patient presentation and initiation of treatment. Evaluating the effect of late presentation would require a different methodological approach such as multi‐state modelling, and an additional information on patients' status upon hospital arrival and the progression of the disease. Fifth, this study did not examine the differential impact of ICT receipt on survival. The Kaplan–Meier curves suggest an inferior survival of Q5 compared to Q1 (Figure S4). Lastly, the national datasets may be susceptible to underdiagnosis or missing treatment records during the pandemic because of the health service crisis. The completeness of the SACT dataset improved over time (Table S11), and the distribution of baseline characteristics among patients with missing treatment data remained stable across the study period (Table S12).

## CONCLUSION

Deprivation is associated with inequality in ICT receipt among patients with AML in England. Deprivation‐related inequality is driven by service variability among trusts, which stems from factors like institutional culture, workforce capacity and resource availability. A more in‐depth study that focuses on decision‐making processes and service capacity at NHS Trusts may help in quantifying the variations of AML treatment based on socioeconomic factors and understanding the cause of its variability.

## AUTHOR CONTRIBUTIONS

**Koki Shimizu:** Conceptualization; methodology; investigation; formal analysis; funding acquisition; visualization; writing – original draft. **Mari Kajiwara Saito:** Conceptualization; methodology; supervision; funding acquisition; writing – review and editing. **Camille Maringe:** Conceptualization; methodology; data curation; supervision; funding acquisition; writing – review and editing. **Yasushi Miyazaki:** Conceptualization; methodology; supervision; funding acquisition; writing – review and editing. **Bernard Rachet:** Conceptualization; methodology; data curation; supervision; funding acquisition; writing – review and editing.

## FUNDING INFORMATION

This study was funded by the Nagasaki University WISE Programme Research Grant and Cancer Research UK programme (grant no. EPNCZS34). The funders had no role in study design, data collection and analysis, decision to publish or preparation of the manuscript.

## CONFLICT OF INTEREST STATEMENT

All authors have no conflicts of interest.

## Supporting information


**Figure S1:** The flow chart of data linkage process to retrieve treatment information from the four national datasets (SACT/HES/NCRD/RTDS).
**Figure S2:** Distribution of days between the date of AML diagnosis and the initiation of first intensive chemotherapy (ICT) (*N* = 5190).
**Figure S3:** Prevalence of ICT receipt by age in male and female patients with and without comorbidities.
**Figure S4:** The overall survival of patients in Q1 and Q5 by age group and treatment intensity estimated by the Kaplan–Meier method.
**Table S1:** The list of the ICD‐O‐3 morphology codes and descriptions of acute myeloid leukaemia.
**Table S2:** The list of the ICD‐O‐3 morphology codes and the ICD‐10 codes used to identify antecedent haematological disorders in the NCRD and the HES.
**Table S3:** The list of the ICD‐O‐3 morphology codes, the ICD‐10 codes and the OPCS‐4 codes used to identify previous chemotherapy and radiotherapy procedures in the NCRD, the HES and the RTDS.
**Table S4:** Treatment intensity for chemotherapy regimens in the SACT.
**Table S5:** The list of the ICD‐10 codes and the OPCS‐4 codes used to identify treatment information from the HES.
**Table S6:** Treatment intensity for chemotherapy regimens in the NCRD.
**Table S7:** The list of the ICD‐10 codes used to identify comorbidities in the NCRD and the HES.
**Table S8:** The proportion of patients who initiated treatment at intensive trusts by age group and deprivation.
**Table S9:** Baseline characteristics of patients who had information of the initial treatment trust, stratified by the type of trust (*N* = 9845).
**Table S10:** Difference in prevalence of ICT receipt among patients with AML, in ages between 25 and 80, diagnosed in England between 2015 and 2022, limited to those with information of performance status (*N* = 5292).†
**Table S11:** The number of patients who had a record of chemotherapy between −2 months and 12 months of AML diagnosis in the SACT.
**Table S12:** Baseline characteristics of patients who did not have treatment record in the SACT by year (*N* = 3046).
**Appendix S1.** Review of systemic anti‐cancer therapy treatments.

## Data Availability

Datasets in this study are available for specific research purposes through application to Public Health England. Additional analyses may be requested from the corresponding author.
