# Supplementary material for: Socioeconomic inequality in treatment of acute myeloid leukaemia (AML) in England, 2015–2022: A population‐based study
Source: Br J Haematol. 2026 Jul 14;209(3):1128–35. doi: 10.1111/bjh.70695 (PMC13570205; doi:10.1111/bjh.70695)
Supplement: Supplementary file 1 [file BJH-209-1128-s001.docx]

**Socioeconomic inequalities in treatment of acute myeloid leukaemia in England, 2015–2022: a population-based study**

**Supplementary material**

Table of contents

[Table S1: The list of the ICD-O-3 morphology codes and descriptions of acute myeloid leukaemia	2](#_Toc231910589)

[Table S2: The list of the ICD-O-3 morphology codes and the ICD-10 codes used to identify antecedent haematological disorders in the NCRD and the HES	3](#_Toc231910590)

[Table S3: The list of the ICD-O-3 morphology codes, the ICD-10 codes, and the OPCS-4 codes used to identify previous chemotherapy and radiotherapy procedures in the NCRD, the HES, and the RTDS	4](#_Toc231910591)

[Appendix 1: Review of systemic anti-cancer therapy treatments	6](#_Toc231910592)

[Figure S1: The flow chart of data linkage process to retrieve treatment information from the four national datasets (SACT/HES/NCRD/RTDS)	7](#_Toc231910593)

[Table S4: Treatment intensity for chemotherapy regimens in the SACT	8](#_Toc231910594)

[Table S5: The list of the ICD-10 codes and the OPCS-4 codes used to identify treatment information from the HES	18](#_Toc231910595)

[Table S6: Treatment intensity for chemotherapy regimens in the NCRD	21](#_Toc231910596)

[Figure S2: Distribution of days between the date of AML diagnosis and the initiation of first intensive chemotherapy (ICT) (N=5,190)	22](#_Toc231910597)

[Table S7: The list of the ICD-10 codes used to identify comorbidities in the NCRD and the HES	23](#_Toc231910598)

[Figure S3: Prevalence of ICT receipt by age in male and female patients with and without comorbidities	24](#_Toc231910599)

[Table S8: The proportion of patients who initiated treatment at intensive Trusts by age group and deprivation	25](#_Toc231910600)

[Table S9: Baseline characteristics of patients that had information of the initial treatment Trust, stratified by the type of Trust (N=9,845)	26](#_Toc231910601)

[Table S10: Difference in prevalence of ICT receipt among patients with AML, in ages between 25 and 80, diagnosed in England between 2015 and 2022, limited to those with information of performance status (N=5,292) †	27](#_Toc231910602)

[Figure S4: The overall survival of patients in Q1 and Q5 by age group and treatment intensity estimated by the Kaplan-Meier method	28](#_Toc231910603)

[Table S11: The number of patients that had a record of chemotherapy between –2 months to 12 months of AML diagnosis in the SACT	29](#_Toc231910604)

[Table S12: Baseline characteristics of patients that did not have treatment record in the SACT by year (N=3,046)	30](#_Toc231910605)

# **Table S1: The list of the ICD-O-3 morphology codes and descriptions of acute myeloid leukaemia**

| **Code** | **Description** |
| --- | --- |
| 9840/3 | Acute myeloid leukaemia, M6 type |
| 9861/3 | Acute myeloid leukaemia, NOS |
| 9865/3 | Acute myeloid leukaemia with t(6;9) (p23;q34); DEK-NUP214 |
| 9867/3 | Acute myelomonocytic leukaemia |
| 9869/3 | Acute myeloid leukaemia with inv(3) (q21;q26.2) or t(3.3) (q21;q26.2); RPN1-EVI1 |
| 9870/3 | Acute basophilic leukaemia |
| 9871/3 | Acute myeloid leukaemia with abnormal marrow eosinophils |
| 9872/3 | Acute myeloid leukaemia, minimal differentiation |
| 9873/3 | Acute myeloid leukaemia without maturation |
| 9874/3 | Acute myeloid leukaemia with maturation |
| 9877/3 | Acute myeloid leukaemia with mutated NPM1 |
| 9878/3 | Acute myeloid leukaemia with biallelic mutation of CEBPA |
| 9879/3 | Acute myeloid leukaemia with mutated RUNX1 |
| 9891/3 | Acute monocytic leukaemia |
| 9895/3 | Acute myeloid leukaemia with myelodysplasia-related changes |
| 9896/3 | Acute myeloid leukaemia, t(8;21) (q22;q22) |
| 9897/3 | Acute myeloid leukaemia, 11q23 abnormalities |
| 9898/3 | Myeloid leukaemia associated with Down Syndrome |
| 9910/3 | Acute megakaryoblastic leukaemia |
| 9911/3 | Acute myeloid leukaemia (megakaryoblastic) with t(1;22) (p13;q13); RBM15-MKL1 |
| 9912/3 | Acute myeloid leukaemia with BCR-ABL1 |
| 9930/3 | Myeloid sarcoma |
| 9931/3 | Acute panmyelosis with myelofibrosis |

Abbreviations: ICD-O-3, International Classification Diseases for Oncology 3^rd^ Edition.

# **Table S2: The list of the ICD-O-3 morphology codes and the ICD-10 codes used to identify antecedent haematological disorders in the NCRD and the HES**

| **Data** | **Classification** | **Code** | **Description** |
| --- | --- | --- | --- |
| NCRD | ICD-O-3 | 9945/3 | Chronic myelomonocytic leukaemia |
| NCRD | ICD-O-3 | 9946/3 | Juvenile myelomonocytic leukaemia |
| NCRD | ICD-O-3 | 9950/3 | Polycythaemia vera |
| NCRD | ICD-O-3 | 9960/3 | Myeloproliferative neoplasm |
| NCRD | ICD-O-3 | 9961/3 | Primary myelofibrosis |
| NCRD | ICD-O-3 | 9962/3 | Essential thrombocythaemia |
| NCRD | ICD-O-3 | 9975/3 | Myelodysplastic/myeloproliferative neoplasm, unclassifiable |
| NCRD | ICD-O-3 | 9980/3 | Refractory anaemia |
| NCRD | ICD-O-3 | 9981/3 | Refractory anaemia without sideroblasts |
| NCRD | ICD-O-3 | 9982/3 | Refractory anaemia with sideroblasts |
| NCRD | ICD-O-3 | 9983/3 | Refractory anaemia with excess blasts |
| NCRD | ICD-O-3 | 9984/3 | Refractory anaemia with excess blasts in transformation |
| NCRD | ICD-O-3 | 9985/3 | Refractory cytopenia with multilineage dysplasia |
| NCRD | ICD-O-3 | 9986/3 | Myelodysplastic syndrome with isolated del (5q) |
| NCRD | ICD-O-3 | 9987/3 | Therapy-related myelodysplastic syndrome, NOS |
| NCRD | ICD-O-3 | 9989/3 | Myelodysplastic syndrome, NOS |
| NCRD | ICD-O-3 | 9993/3 | Myelodysplastic syndrome with ring sideroblasts and multilineage dysplasia |
| HES | ICD-10 | C93.1 | Chronic myelomonocytic leukaemia |
| HES | ICD-10 | C93.3 | Juvenile myelomonocytic leukaemia |
| HES | ICD-10 | D45 | Polycythaemia vera |
| HES | ICD-10 | D46.0 | Refractory anaemia without ring sideroblasts |
| HES | ICD-10 | D46.1 | Refractory anaemia with ring sideroblasts |
| HES | ICD-10 | D46.2 | Refractory anaemia with excess blasts |
| HES | ICD-10 | D46.4 | Refractory anaemia, unspecified |
| HES | ICD-10 | D46.5 | Refractory anaemia with multilineage dysplasia |
| HES | ICD-10 | D46.6 | Myelodysplastic syndrome with isolated del (5q) chromosomal abnormality |
| HES | ICD-10 | D46.7 | Other myelodysplastic syndromes |
| HES | ICD-10 | D46.9 | Myelodysplastic syndrome, unspecified |
| HES | ICD-10 | D47.1 | Chronic myeloproliferative disease |
| HES | ICD-10 | D47.3 | Essential thrombocythaemia |
| HES | ICD-10 | D47.4 | Osteomyelofibrosis |
| HES | ICD-10 | D61.9 | Aplastic anaemia, unspecified |

Abbreviations: ICD-10, International Classification Diseases 10^th^ version; ICD-O-3, International Classification Diseases for Oncology 3^rd^ Edition; HES, Hospital Episode Statistics; NCRD, National Cancer Registration Dataset.

# **Table S3: The list of the ICD-O-3 morphology codes, the ICD-10 codes, and the OPCS-4 codes used to identify previous chemotherapy and radiotherapy procedures in the NCRD, the HES, and the RTDS**

| **Dataset** | **Classification** | **Code** | **Description** |
| --- | --- | --- | --- |
| NCRD | ICD-O-3 | 9920 | Therapy-related myeloid neoplasm |
| HES | ICD-10 | Y43.1 | Antineoplastic antimetabolites |
| HES | ICD-10 | Y43.3 | Other antineoplastic drugs |
| HES | ICD-10 | Y63.2 | Overdose of radiation given during therapy |
| HES | ICD-10 | Y84.2 | Radiological procedure and radiotherapy |
| HES | ICD-10 | Z08.2 | Follow-up examination after chemotherapy for malignant neoplasm |
| HES | ICD-10 | Z08.7 | Follow-up examination after combined treatment for malignant neoplasm |
| HES | ICD-10 | Z09.2 | Follow-up examination after chemotherapy for other conditions |
| HES | ICD-10 | Z09.7 | Follow-up examination after combined treatment for other conditions |
| HES | ICD-10 | Z51.0 | Radiotherapy session |
| HES | ICD-10 | Z51.1 | Chemotherapy session for neoplasm |
| HES | ICD-10 | Z51.2 | Other chemotherapy |
| HES | ICD-10 | Z54.2 | Convalescence following chemotherapy |
| HES | ICD-10 | Z92.3 | Personal history of irradiation |
| HES | ICD-10 | Z92.6 | Personal history of chemotherapy for neoplastic disease |
| HES | ICD-10 | Z94.8 | Other transplanted organ and tissue status |
| HES | OPCS-4 | O44.1 | Proton radiotherapy |
| HES | OPCS-4 | O44.8 | Other specified |
| HES | OPCS-4 | O44.9 | Unspecified |
| HES | OPCS-4 | X35.2 | Intravenous chemotherapy |
| HES | OPCS-4 | X37.3 | Intramuscular chemotherapy |
| HES | OPCS-4 | X38.4 | Subcutaneous chemotherapy |
| HES/RTDS | OPCS-4 | X65.1 | Delivery of a fraction of total body irradiation |
| HES/RTDS | OPCS-4 | X65.4 | Delivery of a fraction of external beam radiotherapy |
| HES/RTDS | OPCS-4 | X65.8 | Other specified |
| HES | OPCS-4 | X65.9 | Unspecified |
| HES/RTDS | OPCS-4 | X67.1 | Preparation for intensity modulated radiation therapy |
| HES/RTDS | OPCS-4 | X67.2 | Preparation for total bod irradiation |
| HES/RTDS | OPCS-4 | X67.3 | Preparation for hemi body irradiation |
| HES/RTDS | OPCS-4 | X67.4 | Preparation for simple radiotherapy with imaging and dosimetry |
| HES/RTDS | OPCS-4 | X67.5 | Preparation for simple radiotherapy with imaging and simple calculation |
| HES/RTDS | OPCS-4 | X67.6 | Preparation for superficial radiotherapy with simple calculation |
| HES/RTDS | OPCS-4 | X67.7 | Preparation for complex conformal radiotherapy |
| HES/RTDS | OPCS-4 | X67.8 | Other specified |
| HES/RTDS | OPCS-4 | X67.9 | Unspecified |
| HES | OPCS-4 | X69.1 | Delivery of a fraction of hemi-body radiation |
| HES | OPCS-4 | X69.2 | Delivery of a fraction of total skin radiotherapy |
| HES | OPCS-4 | X69.8 | Other specified |
| HES | OPCS-4 | X69.9 | Unspecified |
| HES | OPCS-4 | X72.2 | Delivery of complex parenteral chemotherapy for neoplasm at first attendance |
| HES | OPCS-4 | X72.3 | Delivery of simple parenteral chemotherapy for neoplasm at first attendance |
| HES | OPCS-4 | X72.4 | Delivery of subsequent element of cycle of chemotherapy for neoplasm |
| HES | OPCS-4 | X72.8 | Other specified |
| HES | OPCS-4 | X72.9 | Unspecified |
| HES | OPCS-4 | X73.1 | Delivery of exclusively oral chemotherapy for neoplasm |
| HES | OPCS-4 | X73.8 | Other specified |
| HES | OPCS-4 | X73.9 | Unspecified |
| HES | OPCS-4 | X74.2 | Cancer supportive drugs Band 1 |
| HES | OPCS-4 | Y90.2 | Radiotherapy NEC |
| HES/RTDS | OPCS-4 | Y91.1 | Megavoltage treatment for complex radiotherapy |
| HES/RTDS | OPCS-4 | Y91.2 | Megavoltage treatment for simple radiotherapy |
| HES/RTDS | OPCS-4 | Y91.3 | Superficial or orthovoltage treatment for radiotherapy |
| HES/RTDS | OPCS-4 | Y91.4 | Megavoltage treatment for adaptive radiotherapy |
| HES/RTDS | OPCS-4 | Y91.5 | Megavoltage treatment for hypofractionated stereotactic radiotherapy |
| **Dataset** | **Classification** | **Code** | **Description** |
| HES/RTDS | OPCS-4 | Y91.6 | Intraoperative electron beam radiotherapy |
| HES/RTDS | OPCS-4 | Y91.7 | Electron beam radiotherapy NEC |
| HES/RTDS | OPCS-4 | Y91.8 | Other specified |
| HES/RTDS | OPCS-4 | Y91.9 | Unspecified |
| HES/RTDS | OPCS-4 | Y92.1 | Technical support for preparation for radiotherapy |
| HES/RTDS | OPCS-4 | Y92.8 | Other specified |
| HES/RTDS | OPCS-4 | Y92.9 | Unspecified |

Abbreviations: HES, Hospital Episode Statistics; ICD-10, International Classification Diseases 10^th^ version; ICD-O-3, International Classification Diseases for Oncology 3^rd^ Edition; NCRD, National Cancer Registration Dataset; OPCS-4, OPCS Classification of Interventions and Procedures version 4; RTDS, Radiotherapy Dataset.

# **Appendix 1: Review of systemic anti-cancer therapy treatments**

The acute myeloid leukaemia (AML) treatment data were extracted from the SACT, the HES, the NCRD, and the RTDS. The treatment information was retrieved from the period between –2 months to 1 year post AML diagnosis. The regimens were first classified if they were key regimens for AML induction or transplant. Subsequently, those regimens that were either relevant to induction or transplant were further categorised into different intensities of treatment; intensive, non-intensive, palliative and unknown.

**Key**

- I = intensively treated
- TI = transplant regimen but must be intensive
- NI = non-intensively treated
- UK = unknown intensity
- E = chemotherapy unrelated to induction or transplantation, or regimen unknown
- P = palliatively treated
- R = radiotherapy

**Algorithm for treatment intensity**

Abbreviations: AML, acute myeloid leukaemia; HES, Hospital Episode Statistics; NCRD, National Cancer Registration Dataset; SACT, Systemic Anti-Cancer Therapy; RTDS, Radiotherapy dataset.

# **Figure S1: The flow chart of data linkage process to retrieve treatment information from the four national datasets (SACT/HES/NCRD/RTDS)**

This flow chart described the data linkage process through which we obtained treatment information from the four national datasets.

^a^ Refer to code lists of Table S4.

^b^ Refer to code lists of Table S5.

^c^ Refer to code lists of Table S6.

^d^ Patients with any record of radiotherapy procedures in the RTDS were considered as those who received radiotherapy treatment.

Abbreviations: AML, acute myeloid leukaemia; HES, Hospital Episode Statistics; ICD-10, International Classification Diseases 10^th^ version; NCRD, National Cancer Registration Dataset; OPCS-4, OPCS Classification of Interventions and Procedures version 4; RTDS, Radiotherapy Dataset; SACT, Systemic Anti-Cancer Therapy.

# **Table S4: Treatment intensity for chemotherapy regimens in the SACT**

Each line of chemotherapy regimen was registered to the SACT with a patient unique identifier, treatment intent, chemotherapy administration date and dosage.

Some centres registered a combination regimen (e.g. DAUNORUBICIN + CYTARABINE) in the SACT, and some centres chose to register drugs separately (e.g. DAUNORUBICIN, CYTARABINE) in the SACT. If a drug appeared alone (e.g. DAUNORUBICIN) in a line, it was possible that a patient either received daunorubicin only or that other drugs were registered in a separate line.

| **Regimen** | **Number of patients that had a record of the regimen** | **Induction** | **Transplant** | **Intent of treatment** | **Drug content or dose** | **Intensity** |
| --- | --- | --- | --- | --- | --- | --- |
| ADE (cytarabine, daunorubicin, etoposide) | 1 | Yes | No | Any |  | I |
| ALEMTUZUMAB | 37 | No | Yes | Any |  | TI |
| ALEMTUZUMAB + BUSULFAN + CYCLOPHOSPHAMIDE | 18 | No | Yes | Any |  | TI |
| ALEMTUZUMAB + BUSULFAN + FLUDARABINE | 43 | No | Yes | Any |  | TI |
| ALEMTUZUMAB + CYCLO + FLUDARABINE | 1 | No | Yes | Any |  | TI |
| ALEMTUZUMAB + CYCLOPHOSPHAMIDE | 4 | No | Yes | Any |  | TI |
| ALEMTUZUMAB + CYCLOPHOSPHAMIDE + FLUDARABINE | 10 | No | Yes | Any |  | TI |
| ALEMTUZUMAB + FLUDARABINE | 3 | No | Yes | Any |  | TI |
| ALEMTUZUMAB + FLUDARABINE + MELPHALAN | 201 | No | Yes | Any |  | TI |
| AMADEUS TRIAL | 86 | No | No |  |  | E |
| AML 18 PILOT TRIAL | 81 | Yes | No | Any |  | I |
| AML ADE (cytarabine, daunorubicin, etoposide) | 1 | Yes | No | Any |  | I |
| AML CYTARABINE | 2 | Yes | No | Curative | Any | I |
| AML CYTARABINE | 2 | Yes | No | Palliative | Any | NI |
| AML INTERIM GUIDELINES FLAG-IDA (fludarabine, cytarabine, G-CSF, idarubicin) | 15 | Yes | No | Any |  | I |
| **Regimen** | **Number of patients** | **Induction** | **Transplant** | **Intent of treatment** | **Drug content or dose** | **Intensity** |
| AML INTERIM GUIDELINES HD CYTARABINE | 2 | No | No |  |  | E |
| AML RELAPSED FLA (fludarabine, cytarabine) – IDARUBICIN | 29 | No | No |  |  | E |
| AML RELAPSED HIGH DOSE CYTARABINE | 9 | No | No |  |  | E |
| AML/MDS RELAPSE AZACITIDINE + VENETOCLAX | 4 | No | No |  |  | E |
| AML15 TRIAL | 1 | Yes | No | Any |  | I |
| AML16 TRIAL | 93 | Yes | No | Curative | Any | I |
| AML16 TRIAL | 19 | Yes | No | Palliative | Any | NI |
| AML16 TRIAL | 10 | Yes | No | Unknown | Drug dosage unknown | UK |
| AML17 TRIAL | 237 | Yes | No | Any | Any | I |
| AML18 TRIAL | 556 | Yes | No | Any | Any | I |
| AML19 TRIAL | 910 | Yes | No | Any | Any | I |
| AMSACRINE | 7 | Yes | No | Any |  | I |
| AMSACRINE + ANTI-THYMOCYTE GLOBULIN (RABBIT) + CYCLOPHOSPHAMIDE + CYTARABINE + FLUDARABINE | 1 | No | Yes | Any |  | TI |
| AMSACRINE + ANTI-THYMOCYTE GLOBULIN + BUSULFAN + CYTARABINE + FLUDARABINE | 14 | No | Yes | Any |  | TI |
| AMSACRINE + BUSULFAN + CYTARABINE + FLUDARABINE | 7 | No | Yes | Any |  | TI |
| AMSACRINE + CYTARABINE | 1 | Yes | No | Any |  | I |
| AMSACRINE + CYTARABINE + ETOPOSIDE | 8 | Yes | No | Any |  | I |
| ANTI-THYMOCYTE GLOBULIN + BUSULFAN + FLUDARABINE | 85 | No | Yes | Any |  | TI |
| ANTI-THYMOCYTE GLOBULIN + BUSULFAN + FLUDARABINE + THIOTEPA | 6 | No | Yes | Any |  | TI |
| **Regimen** | **Number of patients** | **Induction** | **Transplant** | **Intent of treatment** | **Drug content or dose** | **Intensity** |
| ANTI-THYMOCYTE GLOBULIN + CYCLOPHOSPHAMIDE + FLUDARABINE | 1 | No | Yes | Any |  | TI |
| ATEZOLIZUMAB | 1 | No | No |  |  | E |
| ATG | 4 | No | Yes | Any |  | TI |
| ATG + BUSULFAN + FLUDARABINE | 60 | No | Yes | Any |  | TI |
| ATG + BUSULFAN + FLUDARABINE + THIOTEPA | 2 | No | Yes | Any |  | TI |
| AVAPRITINIB | 1 | No | No |  |  | E |
| AZACITIDINE | 1,344 | Yes | No | Any |  | NI |
| AZACITIDINE + VENETOCLAX | 792 | Yes | No | Any |  | NI |
| BMT (bone marrow transplant) | 1 | No | Yes |  |  | TI |
| BORTEZOMIB | 6 | No | No |  |  | E |
| BUSULFAN | 14 | No | Yes | Any |  | TI |
| BUSULFAN + CYCLOPHOSPHAMIDE | 21 | No | Yes | Any |  | TI |
| BUSULFAN + CYCLOPHOSPHAMIDE + FLUDARABINE | 1 | No | Yes |  |  | TI |
| BUSULFAN + CYCLOPHOSPHAMIDE + FLUDARABINE + THIOTEPA | 2 | No | Yes |  |  | TI |
| BUSULFAN + FLUDARABINE | 4 | No | Yes | Any |  | TI |
| BUSULFAN + FLUDARABINE + METHOTREXATE | 10 | No | Yes | Any |  | TI |
| BUSULPHAN + CYCLO | 4 | No | Yes | Any |  | TI |
| BUSULPHAN + FLUDARABINE | 2 | No | Yes | Any |  | TI |
| CABOZANTINIB | 2 | No | No |  |  | E |
| CLADRIBINE | 1 | Yes | No | Any |  | I |
| CLADRIBINE + CYTARABINE + DAUNORUBICIN | 6 | Yes | No | Any |  | I |
| CLOFARABINE | 3 | Yes | No | Any |  | I |
| CLOFARABINE + CYTARABINE | 21 | Yes | No | Any |  | I |
| CLOFARABINE + CYTARABINE + IDARUBICIN | 1 | Yes | No | Any |  | I |
| CLOFARABINE + CYTSARABINE + IDARUBICIN | 2 | Yes | No | Any |  | I |
| CLOFARABINE + DAUNORUBICIN | 1 | Yes | No | Any |  | I |
| **Regimen** | **Number of patients** | **Induction** | **Transplant** | **Intent of treatment** | **Drug content or dose** | **Intensity** |
| COSI TRIAL | 61 | No | Yes | Any |  | TI |
| CYCLOPHOSPHAMIDE | 41 | No | Yes | Any |  | TI |
| CYCLOPHOSPHAMIDE + FLUDARABINE | 109 | No | Yes | Any |  | TI |
| CYCLOPHOSPHAMIDE + FLUDARABINE + MELPHALAN | 1 | No | Yes | Any |  | TI |
| CYCLOPHOSPHAMIDE + FLUDARABINE + THIOTEPA | 12 | No | Yes | Any |  | TI |
| CYCLOPHOSPHAMIDE HIGH DOSE | 3 | No | Yes | Any |  | TI |
| CYTARABINE | 396 | Yes | No | Curative | Any | I |
| CYTARABINE | 18 | Yes | No | Unknown | If cytarabine (≥100mg / administration) is used in the drug group | I |
| CYTARABINE | 499 | Yes | No | Palliative | Any | NI |
| CYTARABINE | 35 | Yes | No | Unknown | If cytarabine (<100mg / administration) is used in the drug group | NI |
| CYTARABINE | 17 | Yes | No | Unknown | Drug dosage unknown | UK |
| CYTARABINE + DAUNORUBICIN | 1,351 | Yes | No | Any |  | I |
| CYTARABINE + DAUNORUBICIN + FLUDARABINE | 3 | Yes | No | Any |  | I |
| CYTARABINE + DAUNORUBICIN + GEMTUZUMAB + MIDOSTAURIN | 1 | Yes | No | Any |  | I |
| CYTARABINE + DAUNORUBICIN + GEMTUZUMAB OZOGAMICIN | 568 | Yes | No | Any |  | I |
| CYTARABINE + ETOPOSIDE | 1 | Yes | No | Any |  | I |
| CYTARABINE + ETOPOSIDE + MITOXANTRONE | 2 | Yes | No | Any |  | I |
| CYTARABINE + FLUDARABINE | 73 | Yes | No | Any |  | I |
| CYTARABINE + FLUDARABINE + IDARUBICIN | 678 | Yes | No | Any |  | I |
| CYTARABINE + GEMTUZUMAB OZOGAMICIN | 34 | Yes | No | Any |  | I |
| CYTARABINE + GLASDEGIB | 1 | Yes | No | Any |  | NI |
| CYTARABINE + IDARUBICIN | 1 | Yes | No | Any |  | I |
|  |  |  |  |  |  |  |
| **Regimen** | **Number of patients** | **Induction** | **Transplant** | **Intent of treatment** | **Drug content or dose** | **Intensity** |
| CYTARABINE + MIDOSTAURIN | 88 | Yes | No | Any |  | I |
| CYTARABINE + MITOXANTRONE | 2 | Yes | No | Any |  | I |
| CYTARABINE + VENETOCLAX | 60 | Yes | No | Any |  | NI |
| CYTARABINE + VOSAROXIN | 1 | Yes | No | Any |  | NI |
| CYTARABINE HIGH DOSE | 952 | No | No | Any |  | E |
| CYTARABINE INTRATHECAL | 26 | No | No |  |  | E |
| DA (daunorubicin, cytarabine) | 905 | Yes | No | Any |  | I |
| DACTINOMYCIN | 2 | No | No |  |  | E |
| DAUNORUBICIN | 32 | Yes | No | Any |  | I |
| DAUNORUBICIN + CYTARABINE + MIDOSTAURIN | 140 | Yes | No | Any |  | I |
| DECITABINE | 2 | Yes | No | Any |  | NI |
| ETOPOSIDE | 10 | No | No |  |  | E |
| ETOPOSIDE + IDARUBICIN | 2 | Yes | No |  |  | I |
| ETOPOSIDE + MITOXANTRONE | 1 | Yes | No |  |  | I |
| FBC (fludarabine, busulfan, cyclophosphamide) | 27 | No | Yes | Any |  | TI |
| FEDRATINIB | 1 | No | No |  |  | E |
| FIGARO TRIAL | 17 | No | Yes | Any |  | TI |
| FLA (fludarabine, cytarabine) | 1 | Yes | No | Any |  | I |
| FLAG (fludarabine, cytarabine, G-CSF) | 40 | Yes | No | Any |  | I |
| FLAG (fludarabine, cytarabine, G-CSF) + IDARUBICIN | 335 | Yes | No | Any |  | I |
| FLUCONAZOLE | 2 | Not chemo | Not chemo |  |  | E |
| FLUDARABINE | 42 | Yes | Yes | Any |  | I |
| FLUDARABINE + MELPHALAN | 38 | No | Yes | Any |  | TI |
| G-CSF | 1 | Not chemo | Not chemo |  |  | E |
| GANETESPIB | 2 | No | No |  |  | E |
| GCLAC (G-CSF, clofarabine, cytarabine) | 1 | Yes | No | Any |  | I |
| GEMTUZUMAB OZOGAMICIN | 89 | Yes | No | Curative |  | I |
| GEMTUZUMAB OZOGAMICIN | 48 | Yes | No | Unknown |  | UK |
| GEMTUZUMAB OZOGAMYCIN | 4 | Yes | No | Curative |  | I |
| GILTERITINIB | 153 | No | No |  |  | E |
|  |  |  |  |  |  |  |
| **Regimen** | **Number of patients** | **Induction** | **Transplant** | **Intent of treatment** | **Drug content or dose** | **Intensity** |
| HYDROCORTISONE | 61 | Not chemo | Not chemo |  |  | E |
| HYDROXYCARBAMIDE | 692 | Yes | No | Any |  | P |
| IDARUBICIN | 36 | Yes | No | Any |  | I |
| IMATINIB | 46 | No | No |  |  | E |
| LENALIDOMIDE | 5 | Yes | No | Any |  | NI |
| LI-1 TRIAL | 45 | Yes | No | Any | Any | NI |
| LIPOSOMAL CYTARABINE | 2 | Yes | No | Curative | Any | I |
| LIPOSOMAL CYTARABINE | 2 | Yes | No | Palliative | Any | NI |
| LIPOSOMAL CYTARABINE | 2 | Yes | No | Unknown | If cytarabine (<100mg / administration) is used in the drug group | NI |
| LIPOSOMAL DAUNORUBICIN | 2 | Yes | No | Any |  | I |
| LIPOSOMAL DAUNORUBICIN + LIPOSOMAL CYTARABINE (VYXEOS) | 250 | Yes | No | Any |  | I |
| MA (liposomal mitoxantrone, cytarabine) | 13 | Yes | No | Any |  | I |
| MACE (amsacrine, cytarabine, etoposide) | 4 | Yes | No | Any |  | I |
| MAGNESIUM ASPARTATE | 1 | Not chemo | Not chemo |  |  | E |
| MELPHALAN | 14 | Yes | No | Any |  | P |
| MERCAPTOPURINE | 2 | Yes | No | Any |  | P |
| MESNA | 5 | Not chemo | Not chemo |  |  | E |
| METHOTREXATE | 71 | No | No |  |  | E |
| METHOTREXATE INTRATHECAL | 12 | No | No |  |  | E |
| MIDAC (mitoxantrone, cytarabine) | 3 | Yes | No | Any |  | I |
| MIDOSTAURIN | 297 | No | No |  |  | E |
| MITOXANTRONE | 16 | Yes | No | Any |  | I |
| NOT CHEMO | 228 | No | No |  |  | E |
| NOT MATCHED | 15 | No | No |  |  | E |
| PILOT TRIAL | 137 | Yes | Yes | Any | Any | I |
| QUIZARTINIB | 17 | No | No |  |  | E |
| RAVVA TRIAL | 11 | Yes | No | Any | Any | NI |
| RITUXIMAB | 160 | No | No |  |  | E |
| RIUXIMAB + VENETOCLAX | 3 | No | No |  |  | E |
| RITUXIMAB BIOSIMILAR (RIXATHON) | 1 | No | No |  |  | E |
| **Regimen** | **Number of patients** | **Induction** | **Transplant** | **Intent of treatment** | **Drug content or dose** | **Intensity** |
| RITUXIMAB BIOSIMILAR (TRUXIMA) | 8 | No | No |  |  | E |
| ROMAZA TRIAL | 1 | Yes | No | Any | Any | NI |
| RUXOLITINIB | 42 | Yes | No | Any |  | NI |
| SORAFENIB | 36 | No | No |  |  | E |
| STEROID | 13 | Not chemo | Not chemo |  |  | E |
| TRANSPLANT ALEMTUZUMAB | 15 | No | Yes | Any |  | TI |
| TRANSPLANT ALEMTUZUMAB + BUSULFAN + CYCLOPHOSPHAMIDE | 11 | No | Yes | Any |  | TI |
| TRANSPLANT ALEMTUZUMAB + BUSULFAN + FLUDARABINE | 11 | No | Yes | Any |  | TI |
| TRANSPLANT ALEMTUZUMAB + CYCLOPHOSPHAMIDE (+/- TBI) | 10 | No | Yes | Any |  | TI |
| TRANSPLANT ALEMTUZUMAB + CYCLOPHOSPHAMIDE + FLUDARABINE (+/- TBI) | 10 | No | Yes | Any |  | TI |
| TRANSPLANT ALEMTUZUMAB + CYCLOPHOSPHAMIDE + METHOTREXATE (+/- TBI) | 1 | No | Yes | Any |  | TI |
| TRANSPLANT ALEMTUZUMAB + FLUDARABINE + MELPHALAN | 224 | No | Yes | Any |  | TI |
| TRANSPLANT ALEMTUZUMAB + FLUDARABINE + TREOSULFAN | 1 | No | Yes | Any |  | TI |
| TRANSPLANT AMSACRINE + ANTI-THYMOCYTE GLOBULIN + BUSULFAN + CYCLOPHOSPHAMIDE + CYTARABINE + FLUDARABINE | 1 | No | Yes | Any |  | TI |
| TRANSPLANT AMSACRINE + ANTI-THYMOCYTE GLOBULIN + BUSULFAN + CYTARABINE + FLUDARABINE | 1 | No | Yes | Any |  | TI |
| **Regimen** | **Number of patients** | **Induction** | **Transplant** | **Intent of treatment** | **Drug content or dose** | **Intensity** |
| TRANSPLANT AMSACRINE + ANTI-THYMOCYTE GLOBULIN + CYCLOPHOSPHAMIDE + CYTARABINE + FLUDARABINE | 14 | No | Yes | Any |  | TI |
| TRANSPLANT AMSACRINE + CYCLOPHOSPHAMIDE + CYTARABINE + FLUDARABINE | 1 | No | Yes | Any |  | TI |
| TRANSPLANT AMSACRINE + CYTARABINE | 2 | No | Yes | Any |  | TI |
| TRANSPLANT ANTI-THYMOCYTE GLOBULIN (RABBIT) + BUSULFAN + FLUDARABINE + THIOTEPA | 2 | No | Yes | Any |  | TI |
| TRANSPLANT ANTI-THYMOCYTE GLOBULIN + BUSULFAN + CYCLOPHOSPHAMIDE | 1 | No | Yes | Any |  | TI |
| TRANSPLANT ANTI-THYMOCYTE GLOBULIN + BUSULFAN + FLUDARABINE | 12 | No | Yes | Any |  | TI |
| TRANSPLANT ANTI-THYMOCYTE GLOBULIN + BUSULFAN + FLUDARABINE + METHOTREXATE | 16 | No | Yes | Any |  | TI |
| TRANSPLANT ANTI-THYMOCYTE GLOBULIN + BUSULFAN + FLUDARABINE + THIOTEPA | 6 | No | Yes | Any |  | TI |
| TRANSPLANT ANTI-THYMOCYTE GLOBULIN + FLUDARABINE | 1 | No | Yes | Any |  | TI |
| TRANSPLANT ANTI-THYMOCYTE GLOBULIN + FLUDARABINE + METHOTREXATE + TREOSULFAN | 11 | No | Yes | Any |  | TI |
| **Regimen** | **Number of patients** | **Induction** | **Transplant** | **Intent of treatment** | **Drug content or dose** | **Intensity** |
| TRANSPLANT ANTI-THYMOCYTE GLOBULIN + FLUDARABINE + TREOSULFAN | 34 | No | Yes | Any |  | TI |
| TRANSPLANT BUSULFAN + CYCLOPHOSPHAMIDE | 9 | No | Yes | Any |  | TI |
| TRANSPLANT BUSULFAN + CYCLOPHOSPHAMIDE + FLUDARABINE + THIOTEPA | 7 | No | Yes | Any |  | TI |
| TRANSPLANT BUSULFAN + CYCLOPHOSPHAMIDE + METHOTREXATE | 1 | No | Yes | Any |  | TI |
| TRANSPLANT BUSULFAN + FLUDARABINE | 28 | No | Yes | Any |  | TI |
| TRANSPLANT BUSULFAN + FLUDARABINE + MELPHALAN | 1 | No | Yes | Any |  | TI |
| TRANSPLANT CYCLOPHOSPHAMIDE (+/- TBI) | 3 | No | Yes | Any |  | TI |
| TRANSPLANT CYCLOPHOSPHAMIDE + FLUDARABINE (+/- TBI) | 75 | No | Yes | Any |  | TI |
| TRANSPLANT CYCLOPHOSPHAMIDE + FLUDARABINE + TREOSULFAN | 5 | No | Yes | Any |  | TI |
| TRANSPLANT FLUDARABINE + MELPHALAN | 17 | No | Yes | Any |  | TI |
| TRANSPLANT FLUDARABINE + METHOTREXATE | 1 | No | Yes | Any |  | TI |
| TRANSPLANT FLUDARABINE + TREOSULFAN | 4 | No | Yes | Any |  | TI |
| TRIAL | 200 | Yes | Yes | Curative | Any | I |
| TRIAL | 56 | Yes | Yes | Palliative | Any | NI |
| TRIAL | 49 | Yes | Yes | Unknown |  | UK |
| TRIAL UNSPECIFIED | 224 | Yes | Yes | Curative | Any | I |
| TRIAL UNSPECIFIED | 65 | Yes | Yes | Palliative | Any | NI |
| TRIAL UNSPECIFIED | 61 | Yes | Yes | Unknown |  | UK |
| TRIPLE INTRATHECAL | 23 | No | No |  |  | E |
| VENETOCLAX | 106 | Yes | No | Any |  | NI |
| ZOLEDRONIC ACID | 1 | Not chemo | Not chemo |  |  | E |

Abbreviations: SACT, Systemic Anti-Cancer Therapy.

# **Table S5: The list of the ICD-10 codes and the OPCS-4 codes used to identify treatment information from the HES**

| **Data** | **Source** | **Code** | **Description** | **Intensity** |
| --- | --- | --- | --- | --- |
| HES | ICD-10 | Y43.1 | Antineoplastic antimetabolites | E |
| HES | ICD-10 | Y43.3 | Other antineoplastic drugs | E |
| HES | ICD-10 | Y44.6 | Natural blood and blood products | P |
| HES | ICD-10 | Y63.2 | Overdose of radiation given during therapy | R |
| HES | ICD-10 | Y84.2 | Radiological procedure and radiotherapy | R |
| HES | ICD-10 | Z03.1 | Observation for suspected malignant neoplasm | P |
| HES | ICD-10 | Z03.8 | Observation for other suspected diseases and conditions | P |
| HES | ICD-10 | Z03.9 | Observation for suspected disease or condition, unspecified | P |
| HES | ICD-10 | Z08.2 | Follow-up examination after chemotherapy for malignant neoplasm | E |
| HES | ICD-10 | Z08.7 | Follow-up examination after combined treatment for malignant neoplasm | E |
| HES | ICD-10 | Z08.8 | Follow-up examination after other treatment for malignant neoplasm | P |
| HES | ICD-10 | Z08.9 | Follow-up examination after unspecified treatment for malignant neoplasm | P |
| HES | ICD-10 | Z09.2 | Follow-up examination after chemotherapy for other conditions | E |
| HES | ICD-10 | Z09.7 | Follow-up examination after combined treatment for other conditions | E |
| HES | ICD-10 | Z09.8 | Follow-up examination after other treatment for other conditions | P |
| HES | ICD-10 | Z09.9 | Follow-up examination after unspecified treatment for other conditions | P |
| HES | ICD-10 | Z51.0 | Radiotherapy session | R |
| HES | ICD-10 | Z51.1 | Chemotherapy session for neoplasm | E |
| HES | ICD-10 | Z51.2 | Other chemotherapy | E |
| HES | ICD-10 | Z51.3 | Blood transfusion | P |
| HES | ICD-10 | Z51.5 | Palliative care | P |
| HES | ICD-10 | Z53.1 | Procedure not carried out because of patient’s decision for reasons of belief and group pressure | P |
| HES | ICD-10 | Z54.2 | Convalescence following chemotherapy | E |
| HES | ICD-10 | Z92.3 | Personal history of irradiation | R |
| HES | ICD-10 | Z92.6 | Personal history of chemotherapy for neoplastic disease | E |
| HES | ICD-10 | Z94.8 | Other transplanted organ and tissue status | E |
| HES | OPCS-4 | A54.2 | Injection of therapeutic substance into cerebrospinal fluid | E |
| HES | OPCS-4 | O44.1 | Proton radiotherapy | R |
| HES | OPCS-4 | O44.8 | Other specified | R |
| HES | OPCS-4 | O44.9 | Unspecified | R |
| HES | OPCS-4 | W34.2 | Allograft of bone marrow NEC | I |
| HES | OPCS-4 | W34.3 | Allograft of bone marrow from sibling donor | I |
| HES | OPCS-4 | W34.4 | Allograft of bone marrow from matched unrelated donor | I |
| HES | OPCS-4 | W34.5 | Allograft of bone marrow from haploidentical donor | I |
| HES | OPCS-4 | W34.6 | Allograft of bone marrow from unmatched unrelated donor | I |
| HES | OPCS-4 | W99.1 | Allograft of cord blood stem cells to bone marrow | I |
| HES | OPCS-4 | W99.8 | Other specified | I |
| HES | OPCS-4 | W99.9 | Unspecified | I |
| HES | OPCS-4 | X29.2 | Continuous subcutaneous infusion of therapeutic substance NEC | P |
| HES | OPCS-4 | X33.2 | Intravenous blood transfusion of packed cells | P |
| HES | OPCS-4 | X33.3 | Intravenous blood transfusion of platelets | P |
| HES | OPCS-4 | X33.6 | Allogeneic peripheral blood stem cell transplant | I |
| HES | OPCS-4 | X33.8 | Other specified | P |
| HES | OPCS-4 | X33.9 | Unspecified | P |
| HES | OPCS-4 | X35.2 | Intravenous chemotherapy | E |
| HES | OPCS-4 | X37.3 | Intramuscular chemotherapy | E |
| HES | OPCS-4 | X38.4 | Subcutaneous chemotherapy | E |
| HES | OPCS-4 | X38.7 | Subcutaneous injection of haematological growth factor | P |
| HES | OPCS-4 | X65.1 | Delivery of a fraction of total body irradiation | R |
| HES | OPCS-4 | X65.4 | Delivery of a fraction of external beam radiotherapy NEC | R |
| HES | OPCS-4 | X65.8 | Other specified | R |
| HES | OPCS-4 | X65.9 | Unspecified | R |
| HES | OPCS-4 | X67.1 | Preparation for intensity modulated radiation therapy | R |
| HES | OPCS-4 | X67.2 | Preparation for total body irradiation | R |
| HES | OPCS-4 | X67.3 | Preparation for hemi body irradiation | R |
| **Data** | **Source** | **Code** | **Description** | **Intensity** |
| HES | OPCS-4 | X67.4 | Preparation for simple radiotherapy with imaging and dosimetry | R |
| HES | OPCS-4 | X67.5 | Preparation for simple radiotherapy with imaging and simple calculation | R |
| HES | OPCS-4 | X67.6 | Preparation for superficial radiotherapy with simple calculation | R |
| HES | OPCS-4 | X67.7 | Preparation for complex conformal radiotherapy | R |
| HES | OPCS-4 | X67.8 | Other specified | R |
| HES | OPCS-4 | X67.9 | Unspecified | R |
| HES | OPCS-4 | X69 | Other radiotherapy | R |
| HES | OPCS-4 | X69.1 | Delivery of a fraction of hemi-body radiation | R |
| HES | OPCS-4 | X69.2 | Delivery of a fraction of total skin radiotherapy | R |
| HES | OPCS-4 | X69.8 | Other specified | R |
| HES | OPCS-4 | X69.9 | Unspecified | R |
| HES | OPCS-4 | X70.1 | Procurement of drugs for chemotherapy for neoplasm for regimens in Band 1 | E |
| HES | OPCS-4 | X70.2 | Procurement of drugs for chemotherapy for neoplasm for regimens in Band 2 | E |
| HES | OPCS-4 | X70.3 | Procurement of drugs for chemotherapy for neoplasm for regimens in Band 3 | E |
| HES | OPCS-4 | X70.4 | Procurement of drugs for chemotherapy for neoplasm for regimens in Band 4 | E |
| HES | OPCS-4 | X70.5 | Procurement of drugs for chemotherapy for neoplasm for regimens in Band 5 | E |
| HES | OPCS-4 | X70.8 | Other specified | E |
| HES | OPCS-4 | X70.9 | Unspecified | E |
| HES | OPCS-4 | X71.1 | Procurement of drugs for chemotherapy for neoplasm for regimens in Band 6 | E |
| HES | OPCS-4 | X71.2 | Procurement of drugs for chemotherapy for neoplasm for regimens in Band 7 | E |
| HES | OPCS-4 | X71.3 | Procurement of drugs for chemotherapy for neoplasm for regimens in Band 8 | E |
| HES | OPCS-4 | X71.4 | Procurement of drugs for chemotherapy for neoplasm for regimens in Band 9 | E |
| HES | OPCS-4 | X71.5 | Procurement of drugs for chemotherapy for neoplasm for regimens in Band 10 | E |
| HES | OPCS-4 | X71.8 | Other specified | E |
| HES | OPCS-4 | X71.9 | Unspecified | E |
| HES | OPCS-4 | X72.1 | Delivery of complex chemotherapy for neoplasm including prolonged infusional treatment at first attendance | E |
| HES | OPCS-4 | X72.2 | Delivery of complex parenteral chemotherapy for neoplasm at first attendance | E |
| HES | OPCS-4 | X72.3 | Delivery of simple parenteral chemotherapy for neoplasm at first attendance | E |
| HES | OPCS-4 | X72.4 | Delivery of subsequent element of cycle of chemotherapy for neoplasm | E |
| HES | OPCS-4 | X72.8 | Other specified | E |
| HES | OPCS-4 | X72.9 | Unspecified | E |
| HES | OPCS-4 | X73.1 | Delivery of exclusively oral chemotherapy for neoplasm | E |
| HES | OPCS-4 | X73.8 | Other specified | E |
| HES | OPCS-4 | X73.9 | Unspecified | E |
| HES | OPCS-4 | X74.2 | Cancer supportive drugs Band 1 | E |
| HES | OPCS-4 | X89.1 | Monoclonal antibodies Band 1 | E |
| HES | OPCS-4 | X89.2 | Monoclonal antibodies Band 2 | E |
| HES | OPCS-4 | X90.3 | Neutropenia drugs Band 1 | E |
| HES | OPCS-4 | X90.7 | Myelodysplastic syndrome drugs Band 1 | E |
| HES | OPCS-4 | Y90.2 | Radiotherapy NEC | R |
| HES | OPCS-4 | Y91.1 | Megavoltage treatment for complex radiotherapy | R |
| HES | OPCS-4 | Y91.2 | Megavoltage treatment for simple radiotherapy | R |
| HES | OPCS-4 | Y91.3 | Superficial or orthovoltage treatment for radiotherapy | R |
| HES | OPCS-4 | Y91.4 | Megavoltage treatment for adaptive radiotherapy | R |
| HES | OPCS-4 | Y91.5 | Megavoltage treatment for hypofractionated stereotactic radiotherapy | R |
| HES | OPCS-4 | Y91.6 | Intraoperative electron beam radiotherapy | R |
| HES | OPCS-4 | Y91.7 | Electron beam radiotherapy NEC | R |
| HES | OPCS-4 | Y91.8 | Other specified | R |
| HES | OPCS-4 | Y91.9 | Unspecified | R |
| HES | OPCS-4 | Y92.1 | Technical support for preparation for radiotherapy | R |
| HES | OPCS-4 | Y92.8 | Other specified | R |
| **Data** | **Source** | **Code** | **Description** | **Intensity** |
| HES | OPCS-4 | Y92.9 | Unspecified | R |

Abbreviations: HES, Hospital Episode Statistics; ICD-10, International Classification Diseases 10^th^ version; OPCS-4, OPCS Classification of Interventions and Procedures version 4.

# **Table S6: Treatment intensity for chemotherapy regimens in the NCRD**

| **Regimen** | **Induction** | **Transplant** | **Intensity** |
| --- | --- | --- | --- |
| AML RELAPSED HIGH DOSE CYTARABINE | No | No | E |
| AZACITIDINE | Yes | No | NI |
| CYTARABINE | Yes | No | UK |
| CYTARABINE + DAUNORUBICIN | Yes | No | I |
| CYTARABINE, DAUNORUBICIN | Yes | No | I |
| CYTARABINE, FLUDARABINE, G-CSF | Yes | No | I |
| DAUNORUBICIN, CYTARABINE | Yes | No | I |
| HYDROXYCARBAMIDE | Yes | No | P |
| IDARUBICIN, CYTARABINE | Yes | No | I |
| MIDOSTAURIN | No | No | E |
| NOT CHEMO | No | No | E |

Abbreviations: NCRD, National Cancer Registration Dataset.

# **Figure S2: Distribution of days between the date of AML diagnosis and the initiation of first intensive chemotherapy (ICT) (N=5,190)**

This histogram described the distribution of days between the date of AML diagnosis and the initiation of first intensive chemotherapy (ICT). A total of 5,190 patients had a record of ICT in the SACT. The days ranged from –1,182 to 2,561 days, with a median of 8 days. Of 5,190 patients, 5,046 patients (97%) initiated ICT in the period between –2 months to 1 year post AML diagnosis.

Abbreviation: AML, acute myeloid leukaemia.

# **Table S7: The list of the ICD-10 codes used to identify comorbidities in the NCRD and the HES**

| **Data** | **Description** | **Codes** |
| --- | --- | --- |
| HES | Myocardial infarction | I21.x, I22.x, I23.x, I25.2 |
| HES | Congestive heart failure | I11.0, I13.0, I13.2, I25.5, I42.0, I42.5-I42.9, I43.x, I50.x, P29.0 |
| HES | Peripheral vascular disease | I70.x, I71.x, I73.1, I73.8, I73.9, I77.1, I79.0, I79.2, K55.1, K55.8, K55.9, Z95.8, Z95.9 |
| HES | Cerebrovascular disease | G45.x, G46.x, H34.0, I60.x–I69.x |
| HES | Hemiplegia or paraplegia | G04.1, G11.4, G80.1, G80.2, G81.x, G82.x, G83.0–G83.4, G83.9 |
| HES | Dementia | F00.x–F03.x, F05.1, G30.x, G31.1 |
| HES | Chronic pulmonary disease | I27.8, I27.9, J40.x–J47.x, J60.x–J67.x, J68.4, J70.1, J70.3 |
| HES | Rheumatologic disease | M05.x, M06.x, M31.5, M32.x–M34.x, M35.1, M35.3, M36.0 |
| HES | Peptic ulcer disease | K25.x–K28.x |
| HES | Diabetes without chronic complications | E10.0, E10.1, E10.6, E10.8, E10.9, E11.0, E11.1, E11.6, E11.8, E11.9, E12.0, E12.1, E12.6, E12.8, E12.9, E13.0, E13.1, E13.6, E13.8, E13.9, E14.0, E14.1, E14.6, E14.8, E14.9 |
| HES | Diabetes with chronic complications | E10.2-E10.5, E10.7, E11.2–E11.5, E11.7, E12.2–E12.5, E12.7, E13.2–E13.5, E13.7, E14.2–E14.5, E14.7 |
| HES | Renal disease | I12.0, I13.1, N03.2–N03.7, N05.2–N05.7, N18.x, N19.x, N25.0, Z49.0–Z49.2, Z94.0, Z99.2 |
| NCRD | Any malignancy, including lymphoma and leukaemia, except malignant neoplasm of skin | C00.x–C26.x, C30.x–C34.x, C37.x–C41.x, C43.x, C45.x–C58.x, C60.x–C76.x, C81.x–C85.x, C88.x, C90.x–C97.x |
| NCRD | Metastatic solid tumour | C77.x–C80.x |
| HES | Mild liver disease | B18.x, K70.0–K70.3, K70.9, K71.3–K71.5, K71.7, K73.x, K74.x, K76.0, K76.2–K76.4, K76.8, K76.9, Z94.4 |
| HES | Moderate or severe liver disease | I85.0, I85.9, I86.4, I98.2, K70.4, K71.1, K72.1, K72.9, K76.5, K76.6, K76.7 |
| HES | AIDS/HIV | B20.x–B24.x |

Abbreviations: AIDS, acquired immunodeficiency syndrome; HES, Hospital Episode Statistics; HIV, human immunodeficiency virus; ICD-10, International Classification Diseases 10^th^ version; NCRD, National Cancer Registration Dataset.

# **Figure S3: Prevalence of ICT receipt by age in male and female patients with and without comorbidities**

(a) (b)

 (c)	(d)

 (e)	(f)

This figure described the adjusted prevalence of ICT receipt across different ages. The least deprived (Q1) was in blue, and the most deprived (Q5) was in red. (a) male patients with no comorbidity, (b) female patients with no comorbidity, (c) male patients with 1 comorbidity, (d) female patients with 1 comorbidity, (e) male patients with ≥2 comorbidities, (f) female patients with ≥2 comorbidities.

Abbreviation: ICT, intensive chemotherapy.

# **Table S8: The proportion of patients who initiated treatment at intensive Trusts by age group and deprivation**

|  | **Age 25–59**  **N (%)** | **Age 60–69**  **N (%)** | **Age 70–80**  **N (%)** |
| --- | --- | --- | --- |
| **Q1 (least deprived)** | 548/574 (95) | 509/554 (92) | 861/1,046 (82) |
| **Q5 (most deprived)** | 679/709 (96) | 427/475 (90) | 465/642 (74) |

# **Table S9: Baseline characteristics of patients that had information of the initial treatment Trust, stratified by the type of Trust (N=9,845)**

|  | **Patients with information of initial treatment Trust N (%)** | **Intensive Trust**  **N (%)** | **Non-intensive Trust**  **N (%)** |
| --- | --- | --- | --- |
| **Patients^a^** | 9,845 | 9,185 (93) | 660 (7) |
| **Sex** |  |  |  |
| Male | 5,670 (58) | 5,266 (57) | 404 (61) |
| Female | 4,175 (42) | 3,919 (43) | 256 (39) |
| **Age (years)** |  |  |  |
| Median (IQR) | 67 (56–74) | 66 (55–73) | 75 (71–78) |
| 25─59 | 3,091 (31) | 3,060 (33) | 31 (5) |
| 60─69 | 2,670 (27) | 2,571 (28) | 99 (15) |
| 70─80 | 4,084 (41) | 3,554 (39) | 530 (80) |
| **Number of comorbidities** |  |  |  |
| 0 | 6,739 (68) | 6,408 (70) | 331 (50) |
| 1 | 1,779 (19) | 1,779 (19) | 167 (25) |
| 2 or more | 998 (11) | 998 (11) | 162 (25) |

^a^ First row of the table in row proportion, and the remaining of the table is in column proportion.

# **Table S10: Difference in prevalence of ICT receipt among patients with AML, in ages between 25 and 80, diagnosed in England between 2015 and 2022, limited to those with information of performance status (N=5,292) †**

|  | **Crude difference in prevalence (%)** | **95% confidence interval (%)** | **p-value** ‡ | **Adjusted difference in prevalence (%)** | **95% confidence interval (%)** | **p-value** ‡ |
| --- | --- | --- | --- | --- | --- | --- |
|  | | | | |  |  |
| **Q1 (least deprived)** | Reference |  | 0·06 | Reference |  | 0·001 |
| **Q2** | 1·2 | (–2·2, 4.6) |  | 0·3 | (–2·1, 2·6) |  |
| **Q3** | 3·8 | (0·8, 6.8) |  | 0·4 | (–1·6, 2·4) |  |
| **Q4** | 2·5 | (–1·1, 6·1) |  | –2·0 | (–4·0, –0·1) |  |
| **Q5 (most deprived)** | 4·2 | (–0·2, 8·6) |  | –4·9 | (–8·3, –1·4) |  |

† We analysed 5,292 patients that had information of the initial treatment Trust and performance status upon initiation of the first chemotherapy. The multivariable GEE model adjusted for age, sex, comorbidity and performance status, and included an interaction between age and deprivation.

‡ P values of joint Wald χ^2^ test.

Abbreviations: AML, acute myeloid leukaemia; GEE, generalised estimating equation; ICT, intensive chemotherapy.

# **Figure S4: The overall survival of patients in Q1 and Q5 by age group and treatment intensity estimated by the Kaplan-Meier method**

(a)	(b)

Log-rank test: p = 0·13

Log-rank test: p = 0·09

(c)	(d)

Log-rank test: p = 0·12

Log-rank test: p = 0·01

(e)	(f)

Log-rank test: p < 0·001

Log-rank test: p = 0·01

This figure described the overall survival of the least deprived patients (Q1) and the most deprived patients (Q5). Q1 was in blue line, and Q5 was in red line. The median follow-up time was 1808 days. (a) patients with ICT in ages 25─59, (b) patients with non-ICT in ages 25─59, (c) patients with ICT in ages 60─69, (d) patients with non-ICT in ages 60─69, (e) patients with ICT in ages 70─80, (f) patients with non-ICT in ages 70─80.

Abbreviation: AML, acute myeloid leukaemia; ICT, intensive chemotherapy.

# **Table S11: The number of patients that had a record of chemotherapy between –2 months to 12 months of AML diagnosis in the SACT**

| **Year** | **Total, N** | **Treatment records present in SACT, N (%)** |
| --- | --- | --- |
| 2015 | 1,425 | 873 (61) |
| 2016 | 1,349 | 854 (63) |
| 2017 | 1,396 | 939 (67) |
| 2018 | 1,255 | 909 (72) |
| 2019 | 1,254 | 923 (74) |
| 2020 | 1,270 | 957 (75) |
| 2021 | 1,247 | 974 (78) |
| 2022 | 1,299 | 1,020 (79) |

Abbreviations: AML, acute myeloid leukaemia; SACT, Systemic Anti-Cancer Therapy.

**Table S12: Baseline characteristics of patients that did not have treatment record in the SACT by year (N=3,046)^a^**

|  | **Total**  **N (%)** | **2015**  **N (%)** | **2016**  **N (%)** | **2017**  **N (%)** | **2018**  **N (%)** | **2019**  **N (%)** | **2020**  **N (%)** | **2021**  **N (%)** | **2022**  **N (%)** |
| --- | --- | --- | --- | --- | --- | --- | --- | --- | --- |
| Patients | 3,046 | 552 | 495 | 457 | 346 | 331 | 313 | 273 | 279 |
| Sex |  |  |  |  |  |  |  |  |  |
| Male | 1,811 (59) | 309 (56) | 288 (58) | 279 (61) | 212 (61) | 220 (66) | 172 (55) | 163 (60) | 168 (60) |
| Female | 1,235 (41) | 243 (44) | 207 (42) | 178 (39) | 134 (39) | 111 (34) | 141 (45) | 110 (40) | 111 (40) |
| Age (years) |  |  |  |  |  |  |  |  |  |
| 25–59 | 495 (16) | 107 (19) | 88 (18) | 85 (19) | 47 (14) | 52 (16) | 41 (13) | 42 (15) | 33 (12) |
| 60–69 | 664 (22) | 132 (24) | 111 (22) | 110 (24) | 66 (19) | 53 (16) | 74 (24) | 66 (24) | 52 (19) |
| 70–80 | 1,887 (62) | 313 (57) | 296 (60) | 262 (57) | 233 (67) | 226 (68) | 198 (63) | 165 (60) | 194 (70) |
| Number of comorbidities |  |  |  |  |  |  |  |  |  |
| 0 | 1,717 (56) | 343 (62) | 289 (58) | 250 (55) | 199 (58) | 176 (53) | 158 (50) | 158 (58) | 144 (52) |
| 1 | 693 (23) | 115 (21) | 114 (23) | 114 (25) | 79 (23) | 73 (22) | 76 (24) | 55 (20) | 67 (24) |
| 2 or more | 636 (21) | 94 (17) | 92 (19) | 93 (20) | 68 (20) | 82 (25) | 79 (25) | 60 (22) | 68 (24) |
| Deprivation |  |  |  |  |  |  |  |  |  |
| Q1 (least deprived) | 578 (19) | 99 (18) | 84 (17) | 80 (18) | 67 (19) | 73 (22) | 67 (21) | 58 (21) | 50 (18) |
| Q2 | 648 (21) | 123 (22) | 106 (21) | 90 (20) | 74 (21) | 75 (23) | 69 (22) | 52 (19) | 59 (21) |
| Q3 | 652 (21) | 134 (24) | 95 (19) | 101 (22) | 74 (21) | 69 (21) | 42 (13) | 64 (23) | 73 (26) |
| Q4 | 599 (20) | 109 (20) | 94 (19) | 99 (22) | 62 (18) | 60 (18) | 69 (22) | 53 (19) | 53 (19) |
| Q5 (most deprived) | 569 (19) | 87 (16) | 116 (23) | 87 (19) | 69 (20) | 54 (16) | 66 (21) | 46 (17) | 44 (16) |

^a^ The table is in column proportion.

Abbreviations: SACT, Systemic Anti-Cancer Therapy.
